# Supplementary material for: Effect of Solvent on Fluorescence Emission from Polyethylene Glycol-Coated Graphene Quantum Dots under Blue Light Illumination
Source: Nanomaterials (Basel). 2021 May 24;11(6):1383. doi: 10.3390/nano11061383 (PMC8225077; doi:10.3390/nano11061383)
Supplement: Supplementary file 1 [file nanomaterials-11-01383-s001.zip › nanomaterials-1211615-supplementary.pdf]

nanomaterials

Article

Effect of Solvent on Fluorescence Emission from Polyethylene Glycol-Coated Graphene Quantum Dots under Blue Light Illumination

Po-Chih Yang [1,*], Yu-Xuan Ting [1], Siyong Gu [2], Yasser Ashraf Gandomi [3], Jianlin Li [4], Chien-Te Hsieh [1,*]

1 Department of Chemical Engineering and Materials Science, Yuan Ze University, Taoyuan 32003, Taiwan; danny0935331003@gmail.com
2 Fujian Provincial Key Laboratory of Functional Materials and Applications, School of Materials Science and Engineering, Xiamen University of Technology, Xiamen 361024, China; gu-siyong@163.com
3 Department of Chemical Engineering, Massachusetts Institute of Technology, Cambridge, MA 02142, USA; ygandomi@mit.edu
4 Electrification and Energy Infrastructure Division, Oak Ridge National Laboratory, Oak Ridge, TN 37831, USA; lij4@ornl.gov
* Correspondence: pcyang@saturn.yzu.edu.tw (P.-C.Y.); cthsieh@saturn.yzu.edu.tw (C.-T.H.)

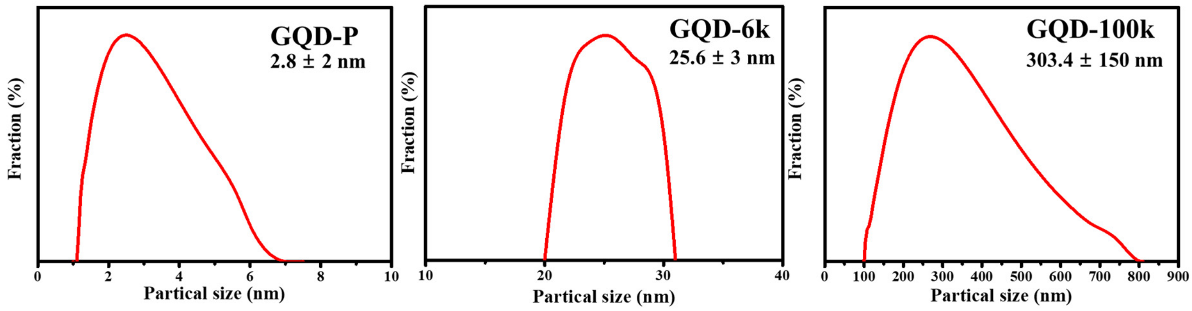

Figure S1. Particle size distributions of different GQD samples determined from DLS technique.

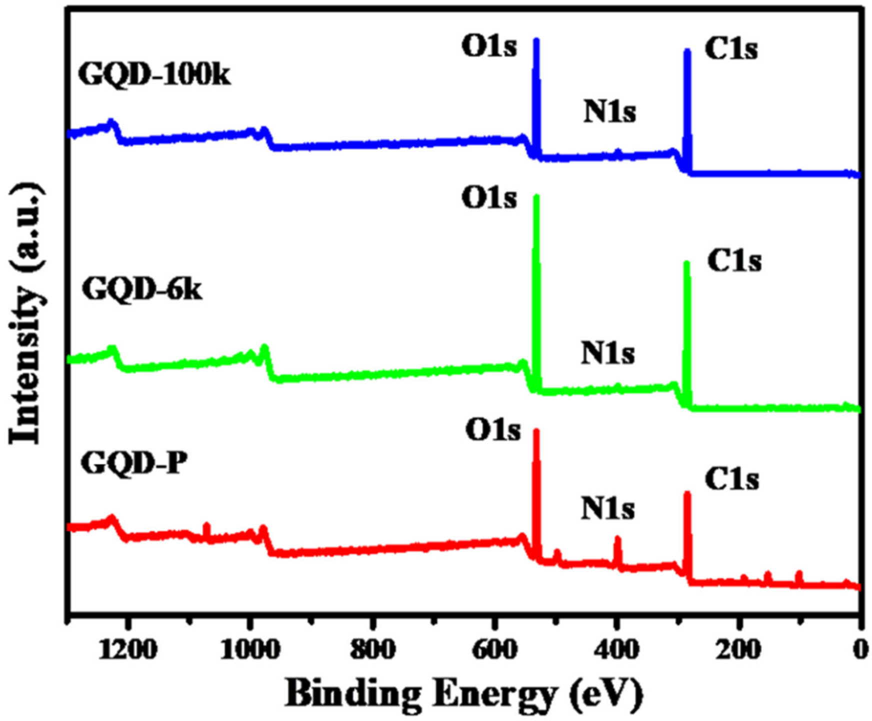

Figure S2. Survey-scan XPS spectra of pristine GQD and PEG-coated GQD cluster samples.

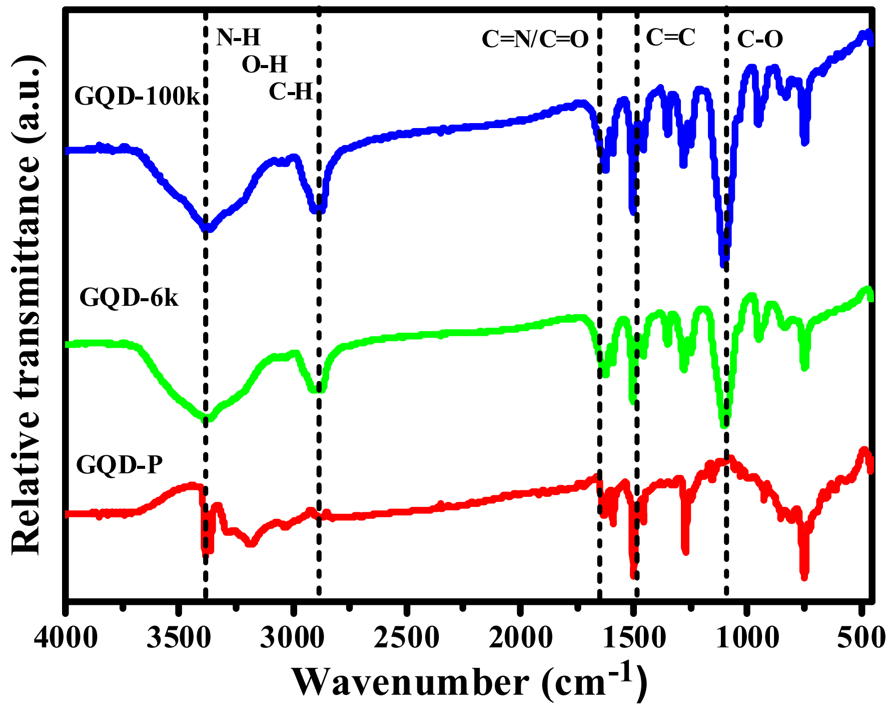

Figure S3. FT-IR spectra of pristine GQD and PEG-coated GQD cluster samples.

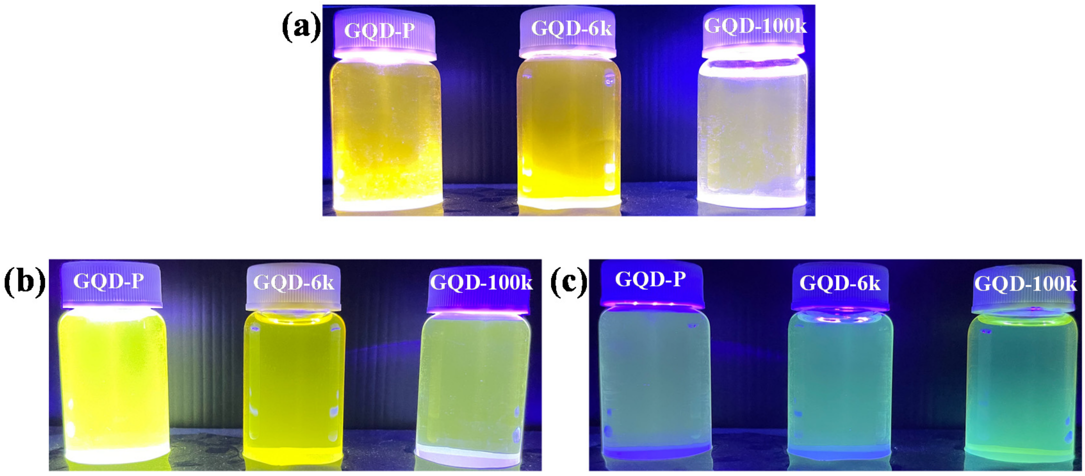

Figure S4. Photographs of different GQD suspensions at 450 nm: **(a)** water, **(b)** ethanol, and **(c)** PGMEA.
